# Effects of different discount levels on healthy products coupled with a healthy choice label, special offer label or both: results from a web-based supermarket experiment

**DOI:** 10.1186/1479-5868-10-59

**Published:** 2013-05-16

**Authors:** Wilma E Waterlander, Ingrid HM Steenhuis, Michiel R de Boer, Albertine J Schuit, Jacob C Seidell

**Affiliations:** 1 Department of Health Sciences and the EMGO Institute for Health and Care Research, Faculty of Earth and Life Sciences, VU University Amsterdam, De Boelelaan 1085, Amsterdam, 1081 HV, The Netherlands; 2 Department of Health Sciences, Community and Occupational Medicine, University Medical Center Groningen, University of Groningen, P.O. Box 30006, Groningen, The Netherlands; 3 National Institute for Public Health and the Environment, P.O. Box 1, Bilthoven, 3720 BA, The Netherlands; 4 Present address: National Institute for Health Innovation, School of Population Health, The University of Auckland – Tamaki Campus, Private Bag 92019, Auckland Mail Centre, Auckland, 1142, New Zealand

**Keywords:** Experiment, Food pricing, Food labelling, Price discounts, Supermarket, Intervention, Public health nutrition, Health promotion, Virtual supermarket

## Abstract

**Background:**

Two strategies commonly recommended to improve population diets include food labels and food taxes/subsidies. The aim of this study was to examine the effects of both strategies separately and in combination.

**Findings:**

An experiment with a 3x3 factorial design was conducted, including: three levels of price reduction (10%; 25%; and 50%) x three labels (‘special offer’, ‘healthy choice’ and ‘special offer & healthy choice’) on healthy foods defined following the Choices front-of-pack nutrition label. N = 109 participants completed the experiment by conducting a typical weekly shop for their household at a three-dimensional web-based supermarket. Data were analysed using analysis of covariance.

Participants receiving a 50% price discount purchased significantly more healthy foods for their household in a typical weekly shop than the 10% discount (+8.7 items; 95%CI = 3.8-13.6) and the 25% discount group (+7.7 items; 95%CI = 2.74 – 12.6). However, the proportion of healthy foods was not significantly higher and the discounts lead to an increased amount of energy purchased. No significant effects of the labels were found.

**Conclusion:**

This study brings some relevant insights into the effects of price discounts on healthier foods coupled with different labels and shows that price effects over shadowed food labels. However, price discounts seem to have ambiguous effects; they do encourage the purchase of healthy products, but also lead to increased energy purchases. More research is needed to examine how pricing strategies can work in directing consumers towards interchanging unhealthier options for healthier alternatives.

## Findings

### Research hypothesis

Two strategies commonly recommended to improve population diets include front-of-pack (FOP) labels and food taxes/subsidies [1,2]. While there is a growing body of evidence on the effects of these strategies, there is a lack of randomized controlled trials (RCT’s) conducted in the major food environment (supermarkets). A recently published review on consumer response to FOP labelling [3] identified only six studies that measured effects of FOP labels on actual food *purchases*[3] and none of these six studies had a controlled experimental design. Similarly, the number of supermarket RCT’s examining food pricing strategies is scarce [4,5]. Supermarket experiments testing food labelling and pricing are particularly important to study cross-price elasticity and/or substitution effects and to measure effects on overall food purchases (e.g., people might spend more money on unhealthy food if healthy food is discounted [6,7] or buy less products with a health logo because they link it with bad taste [8]). Finally, it is worth exploring whether the effects of labels and discounts could be reinforced if they are combined [9]. Here, labels could be used to identify healthier products, but also to highlight a product promotion.

This study aimed to examine the effects of price discounts on healthy foods in combination with signs informing that the product is healthy, discounted or both. It was hypothesized that the most favourable nutrient purchases would be found when combining the greatest discount with a sign explaining that the item is healthy plus discounted [10].

## Methods

**Figure 1:**
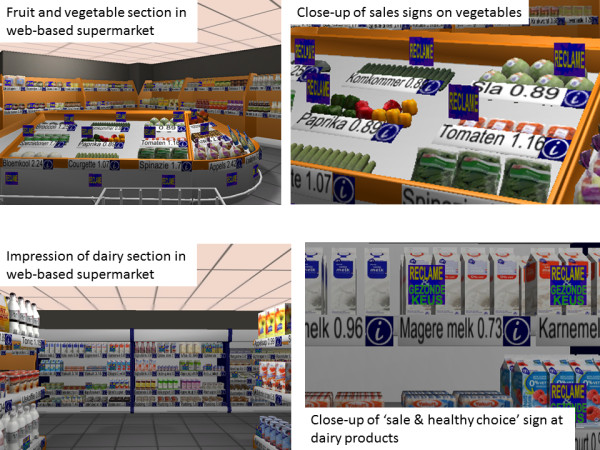
**Impression of the web**-**based supermarket and the used food labels.**

The study was conducted using a three-dimensional (3-D) web-based supermarket (Figure 1). This virtual supermarket was designed to mimic a real-life supermarket and included 512 unique products, modelling the product assortment of a regular supermarket. Further information about the software can be found elsewhere [11,12].

A randomized experiment with three levels of price reduction x three types of labels on healthy foods was conducted. Healthy products were defined following the Choices front-of-pack nutrition label criteria [13] (Table 1). A sample size was determined using delta-values of fruit and vegetable purchases as effect size [14]. It was determined that a sample of n = 108 would be adequate to demonstrate an effect size of .50 (level of significance .05, power > .90, fixed effects, equal sizes in all treatment cells assumed).

**Table 1:** **Number of healthy food products within the 38 food categories in the web-based supermarket**^**a**^

	**Food category**	**Total products (n)**	**Healthy products (n)**
1	Potatoes and potato products	10	7
2	Fruits	10	10
3	Vegetables	41	41
4	Ready to eat meals	19	4
5	Meat/ Fish/ Poultry*	29	13
6	Meat products*	18	4
7	Salads (e.g., crab salad, egg salad, etc.)	8	3
8	Appetizers/ snacks	6	1
9	Cheese	19	3
10	Dairy drinks (e.g., milk, yoghurt drink, etc.)*	15	8
11	Desserts*	21	4
12	(Whipped) cream	5	-
13	Butter	6	2
14	Eggs	2	-
15	Bread*	15	6
16	Pastry	14	4
17	Snacks/ refreshments	12	3
18	Frozen snacks	10	-
19	Ice (cream)	8	1
20	Frozen pastry	2	-
21	Coffee	7	-
22	Evaporated milk/ sugar/ sweeteners	9	2
23	Baking products	13	4
24	Sweet sandwich fillings*	10	3
25	Breakfast products	13	6
26	Pasta/ Rice/ Noodles*	12	4
27	Mixes for sauces	12	1
28	Seasonings	9	1
29	Herbs and spices	10	-
30	Oils/ Sauces and pickles	26	9
31	Soups	12	2
32	Canned foods (excluding fruits and vegetables)	10	3
33	Beverages (excluding soda)	6	3
34	Soda*	24	14
35	Alcoholic beverages	19	-
36	Candy	14	3
37	Chocolate	20	-
38	Crisps/ nuts/ toast	16	3
	**Total**	**512**	**172 (33.6%)**

^a^ Healthy products are defined following the Choices front-of-pack nutrition label criteria which are based on the international WHO recommendations regarding saturated fat, trans fat, sodium, and added sugar [13].

*These product categories were selected for *within* category analysis.

**Figure 2:**
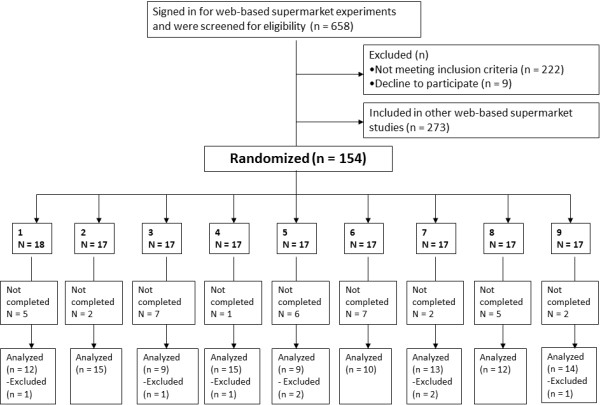
CONSORT flow diagram.

Dutch participants were recruited through newspapers (Figure 2). Inclusion criteria were: being eighteen years or older, speaking Dutch, having an independent household, and having a lower socio-economic status (SES) (having a lower education level or being unemployed). Participants were asked to complete a typical household weekly shop by navigating with a chart between the Virtual Supermarket shelves. Participants received a specific shopping budget, which was calculated based on their household composition, but were not encouraged to spend this entirely. The main outcome measures were: healthy and unhealthy food items (number and proportion); fruit and vegetables (gram); and calories (kcal). As secondary outcome measure we calculated the proportion of healthier products purchased within specific categories (Table 1). Background variables measured included those found in Table 2. Furthermore, we measured: “price perception construct scale items” [15]; self-report index of habit strength [16]; participant’s perception on the quality of the web-based supermarket software; and participant’s notice of prices and their recall of the labels in the web-based supermarket. Answers were all measured on a 7-point Likert Scale.

**Table 2:** Participant characteristics

		**Total n = 109**	**p**^**a**^
		**n (%)**	
Sex	Female	93 (85.3)	.69
Age	18 – 31	18 (16.5)	.15
	32 – 46	56 (51.4)	
	47 – 61	27 (24.8)	
	62 +	8 (7.3)	
Grocery	Totally responsible	68 (62.4)	.18
Responsibility	Largely responsible	24 (22.0)	
	Partly responsible	17 (15.6)	
Education level	Low (primary/ lower secondary)	38 (34.9)	.30
	Medium (higher secondary/ intermediate vocational	58 (53.2)	
	High (higher vocational/ university	13 (11.9)	
Employment status	Employed	38 (34.9)	.38
	Other	71 (65.1)	
Household income	Low (0 – 2000)	32 (29.4)	.09
(€ gross monthly) ^b^	Medium (2000 – 3000)	38 (34.9)	
	High (3000+)	39 (35.8)	
		Mean (SD)	
Household size		2.92 (1.43)	.14
Price perception ^c^		67.93 (9.43)	.73
Habit score ^d.^		49.50 (8.3)	.74
Appreciation score		60.23 (7.37)	.13
Web-based supermarket ^e.^			
Attention to prices in web-based supermarket ^f.^		18.3 (5.3)	.82
Budget in web-based supermarket		70.63 (23.19)	.09
% of budget spent		87.7 (16.0)	.21

Data were measured in 2010 in the Netherlands. Participants included a community sample (n = 109).

^a^ Indicates the p-value for chi^2^ tests and ANOVA analysis comparing the nine research conditions.

^b^ The standard gross monthly income in the Netherlands (2010) was € 2,508 [17].

^c.^ Measured by fifteen items (7-point Likert scale) from the seven “price perception construct scale items” (Lichtenstein et al., 1993).

^d.^ Measured by twelve items (7-point Likert scale) self-report index of habit strength (Verplanken et al., 2003).

^e.^ Measured by eleven items (7-point Likert scale) on the web-based supermarket software.

^f.^ Measured by four items concerning attention to prices in the web-based supermarket (7-point Likert scale).

Differences in food purchases were analysed using two-way factorial ANCOVA models. Model 1 (crude) included the fixed factors level of price discount, type of promotion sign used and the interaction discount x promotion label. Model 2 (fully adjusted) included the fixed terms mentioned above plus the standard factors sex, education, income, purchasing budget in web-based supermarket (low/high) and grocery responsibility and the covariates price perception, habit strength, appreciation of web-based supermarket and notice of prices. Analyses were conducted using SPSS statistical software (version 17.00, SPSS Inc, Chicago, IL).

## Results

N = 109 participants were included in final analysis (Figure 2) (Table 2). 93% of the participants indicated that their experimental purchases aligned with their regular groceries (score ≥5).

The crude models revealed that participants in the 50% discount condition purchased significantly more healthy foods than participants in the 25% or 10% discount condition (Table 3). Likewise, the proportion of healthier products purchased was highest in the 50% discount condition; however differences between groups were not statistically significant. No significant differences were observed in the number of unhealthy foods purchased. Therefore, the total number of foods and total energy purchased was significantly higher in the highest discount condition. Similar results were found when looking within the eight major food categories (Additional file 1). No statistical significant differences between the three label types were found, except for proportion of budget spent (Table 4) (Additional file 2). Similar results were observed in the fully adjusted models. Finally, the interaction discount x promotion sign was not significant at an alpha of 0.10 in any of the models.

**Table 3:** **Effects of varying price *discount *levels on food purchases in the web-based supermarket – results two-way ANCOVA analyses**^**a**^

***Discount***		***10% discount***			***25% discount***		
		**B**	**Lower 95% CI**	**Upper 95% CI**	**B**	**Lower 95% CI**	**Upper 95% CI**
N	*10% discount*	-	-	-	1.81	−3.74	7.35
Unhealthy	*50% discount*	−3.20	−8.42	2.02	−1.39	−6.76	3.98
N Healthy	*10% discount*	-	-	-	−0.44	−5.57	4.70
	*50% discount*	−8.58^**^	−13.4	−3.75	−9.02^***^	−14.0	−4.05
Total items	*10% discount*	-	-	-	1.37	−6.90	9.63
	*50% discount*	−11.8^**^	−19.6	−4.00	−10.4^*^	−18.4	−2.41
Total	*10% discount*	-	-	-	2,899	−4,936	10,733
Calories	*50% discount*	−8,878^*^	−16,258	−1,499	−5,980	−13,566	1,607
N healthy	*10% discount*	-	-	-	−0.41	−4.21	3.38
excl F&V ^b^	*50% discount*	−5.65^**^	−9.22	−2.07	−6.06^**^	−9.73	−2.38
% Healthy	*10% discount*	-	-	-	−2.53	−9.24	4.18
	*50% discount*	−4.02	−10.3	2.30	−6.55‡	−13.1	-.06
% Healthy	*10% discount*	-	-	-	−1.75	−6.46	2.95
excl F&V ^b^	*50% discount*	−2.32	−6.75	2.11	−4.07	−8.63	0.49
Vegetables	*10% discount*	-	-	-	−82.8	−887	721
(gram)	*50% discount*	−1,108	−1,866	−350	−1,191^**^	−1,970	−412
Fruit	*10% discount*	-	-	-	398	−384	1,180
(gram)	*50% discount*	−544	−1,280	193	−146	−903	612
% budget	*10% discount*	-	-	-	−1.34	−8.99	6.31
Spent	*50% discount*	5.52	−1.69	12.7	4.18	−3.23	11.6

Data were measured in 2010 in the Netherlands. Participants included a community sample (n = 109).

^a.^ Results of two-way ANCOVA including the fixed factors level of discount, type of promotion label and the interaction discount x promotion label.

^b.^ Healthy excl F&V means number of healthy products excluding fruits and vegetables.

^*^ significant at p < .05.

^**^ significant at p < .01.

**Table 4:** **Effects of varying price *promotion labels *on food purchases in the web-based supermarket – results two-way ANCOVA analyses**^**a**^

***Type of label***		***Special offer***			***Healthy choice***		
		**B**	**Lower 95% CI**	**Upper 95% CI**	**B**	**Lower 95% CI**	**Upper 95% CI**
N	*Special offer*	-	-	-	4.16	−1.21	9.53
Unhealthy	*Combined label*^*b*^	−1.88	−7.10	3.34	2.28	−3.27	7.82
N Healthy	*Special offer*	-	-	-	1.92	−2.98	6.96
	*Combined label*	2.13	−2.70	6.97	4.12	−1.01	9.26
Total items	*Special offer*	-	-	-	6.15	−1.85	14.2
	*Combined label*	0.25	−7.54	8.04	6.40	−1.86	14.7
Total	*Special offer*	-	-	-	3,013	−4,573	10,600
Calories	*Combined label*	12.4	−7,367	7,392	3,026	−4,808	10,860
N healthy	*Special offer*	-	-	-	2.04	−1.64	5.71
excl F&V ^c^	*Combined label*	1.06	−2.52	4.63	3.09	−0.70	6.89
% Healthy	*Special offer*	-	-	-	−3.36	−9.86	3.13
	*Combined label*	6.00	−0.32	12.32	2.64	−4.07	9.34
% Healthy	*Special offer*	-	-	-	−0.40	−4.95	4.16
excl F&V ^c^	*Combined label*	3.03	−1.40	7.46	2.63	−2.07	7.34
Vegetables	*Special offer*	-	-	-	−219	−998	560
(gram)	*Combined label*	436	−322	1,193	217	−587	1,021
Fruit	*Special offer*	-	-	-	137	−620	895
(gram)	*Combined label*	−60.9	−797	676	76.4	−705	858
% budget	*Special offer*	-	-	-	−6.91	−14.3	0.50
Spent	*Combined label*	8.99^*^	1.78	16.2	2.08	−5.58	9.73

Data were measured in 2010 in the Netherlands. Participants included a community sample (n = 109).

^a.^ Results of two-way ANCOVA including the fixed factors level of discount, type of promotion label and the interaction discount x promotion label.

^b.^ Combined label is ‘special offer & healthy choice’.

^c.^ Healthy excl. means number of healthy products excluding fruits and vegetables.

^*^ significant at p < .05.

^**^ significant at p < .01.

## Discussion

This study in an experimental web-based supermarket examined the effects on food purchases of price discounts on healthy foods in combination with three different labels. Results indicated a positive trend between the proportion of healthier products purchased and higher discounts, however, these differences were not statistically significant. Most importantly, participants significantly increased healthy food purchases due to the price discounts, but did not significantly change the number of *un*healthy foods purchased. Therefore, total energy purchased was significantly higher in the highest discount condition. No significant differences in food purchases were observed between the different label conditions.

An important limitation of this study is the absence of a control condition. Therefore, we were unable to segregate the effects of the price and labeling interventions. Also, it limits the interpretation of the results. Nevertheless, the results from this study bring some relevant new insights, especially since evidence on the effects of price discounts and labels from experimental studies in larger food environments is missing. An important finding was that the price discounts lead to significant higher energy purchases; which is in line with earlier studies [7,18,19] and confirms that it is essential to design price discounts carefully [20]. One possible way to limit extra energy purchases is by restricting the price discounts to fruits and vegetables (opposed to all healthier foods). A recently published pricing experiment revealed that 50% price discounts on fruits and vegetables lead to significantly increased fruit and vegetable purchases and no changes in other food categories [5].

Another relevant finding is that the effects of the price discounts were stronger than the effects of the effects of food labels. For example, in condition one (‘special offer’ & 10% discount) participants purchased on average 21.9 healthy food items; in condition 7 (‘special offer’ & 50% discount) this number was 32.5. Furthermore, our study did not observe differences in food purchases between the label conditions, showing that promotion and health labels had similar effects. While there is much literature on the effects of food labels, most studies to date were limited to consumer understanding instead of effects on purchases [3]. Studies measuring food purchases objectively are vital since understanding a FOP label does not automatically imply that people will change food purchases. One recent study on the effects of FOP traffic-light nutrition labelling on online food purchases using sales data revealed that the traffic light indicators had no influence on sales [21]. Likewise, our study revealed no effects of food labels on food purchases. This has important implications for food labeling policy and shows that FOP labeling alone might not be enough to influence food purchases.

Giessen *et al.* published a study into the effects of calorie information and taxes on high-calorie foods on university student’s lunch decisions. They found that a 25% tax increase was effective to reduce calorie purchases, but that this effect was lowered in the presence of calorie information [22]. The authors therefore argue that it may be more important to communicate calorie information than to tax products. Our study showed no interactions between the price and labelling interventions, and, in contrast to earlier findings, that the sales labels did not upturn the effects of pricing alone. Previous research showed that using the word ‘sale’ beside a price (without actually varying the price) can increase demand by more than 50% [23]. One explanation for the absence of such effects in our study is that our sample size was not specifically powered for these interaction effects. Furthermore, participants might not have felt the necessity to react on the sales labels because they only shopped once in our web-based supermarket and did not consider missing out on future deals [23,24].

## Conclusion

This study brings some relevant insights into the effects of different price discounts on healthier foods coupled with different labels on overall food purchases and forms a valuable basis for future research. Food labels did not seem to have a large impact on food purchases. Price discounts did significantly encourage the purchase of healthy products, but did not discourage the purchase of unhealthy foods and therefore lead to increased energy purchases. More research is needed to unravel how pricing strategies can best be designed to result in overall improved food purchases and what role food labels could have to reach this goal. This research should be specifically aimed at finding ways to direct consumers towards interchanging unhealthier options for healthier alternatives.

## Abbreviations

FOP labels: Front-of-pack labels; RCT: Randomized controlled trial.

## Competing interests

The authors declare that they have no competing interests.

## Authors’ contributions

WEW was responsible for designing the study, data collection, analysis and interpretation of data. This author was involved in drafting the manuscript and has given final approval of the version to be published. IHMS was responsible for the conception and design of this study. This author also revised the manuscript critically for important intellectual content and has given final approval of the version to be published. MRB was responsible for analysis and interpretation of data. This author also revised the manuscript critically for important intellectual content and has given final approval of the version to be published. AJS was responsible for the conception and design of this study. This author also revised the manuscript critically for important intellectual content and has given final approval of the version to be published. JCS was responsible for the conception and design of this study. This author also revised the manuscript critically for important intellectual content and has given final approval of the version to be published. All authors read and approved the final manuscript.

## Supplementary Material

Additional file 1 **Effects of varying price *discount *levels on the percentage of healthy food products purchased within eight different product categories, the Netherlands (2010).** Click here for file

Additional file 2 Effects of varying different promotion labels on the percentage of healthy food products purchased within eight different product categories, the Netherlands (2010). Click here for file
