# Supplementary material for: Effects of different discount levels on healthy products coupled with a healthy choice label, special offer label or both: results from a web-based supermarket experiment
Source: Int J Behav Nutr Phys Act. 2013 May 16;10:59. doi: 10.1186/1479-5868-10-59 (PMC3668240; doi:10.1186/1479-5868-10-59)
Supplement: Additional file 1 — Effects of varying price discount levels on the percentage of healthy food products purchased within eight different product categories, the Netherlands (2010). [file 1479-5868-10-59-S1.docx]

Additional file 1

File format: Table.doc

Title: Effects of varying price *discount* levels on the percentage of healthy food products purchased within eight different product categories, the Netherlands (2010) ^a^

| *Discount* | | *10% discount* | | | *25% discount* | | |
| --- | --- | --- | --- | --- | --- | --- | --- |
|  |  | B | Lower 95% CI | Upper 95% CI | B | Lower 95% CI | Upper 95% CI |
| Meat/Fish/ | *10% discount* | - | - | - | 1.56 | -12.9 | 16.0 |
| Poultry | *50% discount* | -2.94 | -16.5 | 10.6 | -1.38 | -15.2 | 12.5 |
| Meat | *10% discount* | - | - | - | 17.2 | -6.26 | 40.6 |
| products | *50% discount* | -28.6^**^ | -49.1 | -8.04 | -11.4 | -33.0 | 10.1 |
| Dairy | *10% discount* | - | - | - | 18.0^*^ | -0.68 | 36.7 |
| drinks | *50% discount* | -20.9^*^ | -37.5 | -4.20 | -2.83 | -20.2 | 14.5 |
| Desserts | *10% discount* | - | - | - | 1.31 | -21.6 | 24.2 |
|  | *50% discount* | -15.6 | -36.7 | 5.56 | -14.2 | -35.9 | 7.45 |
| Bread | *10% discount* | - | - | - | -6.08 | -22.2 | 10.1 |
|  | *50% discount* | 12.9 | -2.12 | 27.9 | 6.79 | -8.23 | 21.8 |
| Sweet sandwich | *10% discount* | - | - | - | -3.48 | -23.3 | 16.4 |
| fillings | *50% discount* | 2.41 | -15.7 | 20.5 | -1.07 | -19.9 | 17.8 |
| Pasta/ rice/ | *10% discount* | - | - | - | 14.3 | -6.29 | 34.9 |
| noodles | *50% discount* | -6.41 | -25.4 | 12.6 | 7.90 | -11.9 | 27.7 |
| Soda | *10% discount* | - | - | - | -3.30 | -27.8 | 21.2 |
|  | *50% discount* | -9.61 | -31.2 | 12.0 | -12.9 | -25.6 | 9.76 |

*Data were measured in 2010 in the Netherlands. Participants included a community sample (n=109)*

^a.^ Results of two-way ANCOVA including the fixed factors level of discount, type of promotion label and the interaction discount x promotion label and the covariates sex, education, income, purchasing budget in web-based supermarket (low/high), grocery responsibility, price perception, habit strength, appreciation of web-based supermarket and notice of prices

^*^ significant at p <.05

^**^ significant at p<.01
