# Supplementary material for: Effects of different discount levels on healthy products coupled with a healthy choice label, special offer label or both: results from a web-based supermarket experiment
Source: Int J Behav Nutr Phys Act. 2013 May 16;10:59. doi: 10.1186/1479-5868-10-59 (PMC3668240; doi:10.1186/1479-5868-10-59)
Supplement: Additional file 2 — Effects of varying different promotion labels on the percentage of healthy food products purchased within eight different product categories, the Netherlands (2010). [file 1479-5868-10-59-S2.docx]

Additional file 2

Effects of varying different promotion labels on the percentage of healthy food products purchased within eight different product categories, the Netherlands (2010) ^a^

| *Type of label* | | *Special offer* | | | *Healthy choice* | | |
| --- | --- | --- | --- | --- | --- | --- | --- |
|  |  | B | Lower 95% CI | Upper 95% CI | B | Lower 95% CI | Upper 95% CI |
| Meat/Fish/ | *Special offer* | - | - | - | -6.78 | -20.8 | 7.72 |
| Poultry | *Combined label ^b^* | 6.08 | -7.44 | 19.6 | -0.70 | -14.9 | 13.6 |
| Meat | *Special offer* | - | - | - | 5.44 | -15.6 | 26.5 |
| products | *Combined label* | -7.38 | -27.9 | 13.2 | -1.93 | -23.1 | 19.2 |
| Dairy | *Special offer* | - | - | - | -13.7 | -30.3 | 2.98 |
| drinks | *Combined label* | 6.64 | -11.4 | 24.7 | -7.04 | -25.8 | 11.7 |
| Desserts | *Special offer* | - | - | - | -11.5 | -33.8 | 10.8 |
|  | *Combined label* | 6.27 | -14.9 | 27.5 | -5.27 | -27.8 | 17.3 |
| Bread | *Special offer* | - | - | - | 1.61 | -14.1 | 17.3 |
|  | *Combined label* | 6.32 | -8.53 | 21.2 | 7.93 | -8.10 | 24.0 |
| Sweet sandwich | *Special offer* | - | - | - | 0.95 | -18.0 | 19.9 |
| fillings | *Combined label* | -10.2 | -28.6 | 8.18 | -9.28 | -28.7 | 10.1 |
| Pasta/ rice/ | *Special offer* | - | - | - | 9.38 | -10.3 | 29.1 |
| noodles | *Combined label* | -3.53 | -23.2 | 16.1 | 5.85 | -14.5 | 26.2 |
| Soda | *Special offer* | - | - | - | 4.34 | -18.6 | 27.2 |
|  | *Combined label* | 1.65 | -20.1 | 23.4 | 5.99 | -17.6 | 29.6 |

*Data were measured in 2010 in the Netherlands. Participants included a community sample (n=109)*

^a.^ Results of two-way ANCOVA including the fixed factors level of discount, type of promotion label and the interaction discount x promotion label and the covariates sex, education, income, purchasing budget in web-based supermarket (low/high), grocery responsibility, price perception, habit strength, appreciation of web-based supermarket and notice of prices

^b.^ Combined label is ‘special offer & healthy choice’
